# Effects of nail softness and stiffness with distance running shoes on ground reaction forces and vertical loading rates in male elite long-distance runners with pronated feet

**DOI:** 10.1186/s13102-021-00352-7

**Published:** 2021-10-09

**Authors:** Amir Ali Jafarnezhadgero, Ehsan Fakhri, Urs Granacher

**Affiliations:** 1 Department of Sport Managements and Biomechanics, Faculty of Educational Sciences and Psychology, University of Mohaghegh Ardabili, Ardabil, Iran; 2 Division of Training and Movement Sciences, Research Focus Cognition Sciences, University of Potsdam, Potsdam, Germany

**Keywords:** Flat feet, Ground reaction force, Footwear

## Abstract

**Background:**

To improve propulsion during running, athletes often wear spike shoes designed for training and/or competition. Running with spike shoes may cause pain and/or injuries. To address this problem, a modified spike shoe was tested. This study aimed to evaluate the effects of running with dual-versus single-stiffness spike running shoes on running mechanics in long-distance runners with pronated feet.

**Methods:**

Sixteen male elite (national competitive level) runners (5000 or 10,000 m) aged 28.2 ± 2.5 years with pronated feet volunteered to participate in this study. To be included, participants had to have achieved personal best race times over 5- and/or 10-km races under 17 or 34 min during official running competitions. All participants were heel strikers and had a history of 11.2 ± 4.2 years of training. For the assessment of running kinetics, a force plate was imbedded into a walkway. Running kinematics were recorded using a Vicon-motion-capture system. Nike Zoom Rival shoes (Nike, Nike Zoom Rival, USA) were selected and adapted according to spike softness and stiffness. Participants ran at a constant speed of ~4.0 m/s across the walkway with both shoe conditions in randomized order. Six trials were recorded per condition. The main outcomes included peak ground reaction forces and their time-to-peak, average and instantaneous vertical loading rates, free moments, and peak ankle eversion angles.

**Results:**

Paired t-tests revealed significantly lower lateral (*p* = 0.021, d = 0.95) and vertical (*p* = 0.010, d = 1.40) forces at heel contact during running with dual-stiffness spike shoes. Running with dual-stiffness spike shoes resulted in a significantly longer time-to-peak vertical (*p* = 0.004, d = 1.40) force at heel contact. The analysis revealed significantly lower average (*p* = 0.005, d = 0.46) and instantaneous (*p* = 0.021, d = 0.49) loading rates and peak negative free moment amplitudes (*p* = 0.016, d = 0.81) when running with dual-stiffness spike shoes. Finally, significantly lower peak ankle eversion angles were observed with dual-stiffness spike shoes (*p* < 0.001, d = 1.29).

**Conclusions:**

Running in dual- compared with single-stiffness spike distance running shoes resulted in lower loading rates, free moment amplitudes, and peak ankle eversion angles of long-distance runners with pronated feet.

## Background

In recent years, running has become increasingly popular as a recreational and competitive exercise activity. Previous studies revealed that elite long-distance runners perform workloads of 150–260 km per week during a regular season [1–4]. Running training is often periodized across the season and involves mostly medium to high exercise intensities at or slightly below race pace [5]. High intensity interval training and competition over 5000 and 10,000 m is mostly performed on a track using spike shoes [5].

The main function of spiked shoes is to increase friction force between the shoe and the track, to improve propulsion during running [6]. There is information in the literature [7] that competitive long-distance runners (5000 or 10,000 m) wear spike running shoes approximately three hours during daily training and four days a week [7]. Compared to regular running shoes, spike shoes have less cushioning and a thinner heel to reduce the mass of the shoe [8]. Logan et al. [8] examined differences in ground reaction forces (GRFs) when running in distance spike shoes versus regular running shoes (without any spikes) in intercollegiate distance runners. The study demonstrated that loading rates (⁓ 53%), peak vertical impact forces (⁓ 29%) and peak braking forces (⁓ 31%) were significantly greater in spiked compared with regular running shoes [8]. However, while spike running shoes have benefits in terms of propulsion, they also produce negative side effects with regards to an increased injury risk. The traditional or single-stiffness spike running shoe has been designed for runners with neutral feet who run on the forefoot in plantar flexed position [7]. Yet, single-stiffness spike shoes appear not to be well-suited for runners with pronated feet because they cannot counteract the mechanical malalignment caused by foot excessive pronation during running. In clinical practice, pronated feet are classified during static standing tests as a specific type of foot posture and are generally characterized by an everted rearfoot, abducted forefoot and a lower medial longitudinal arch [9]. There is evidence that foot pronation (i.e., severe ankle eversion) is a risk factor for running-related injuries [10], especially when using neutral footwear [11].

**Fig. 1:**
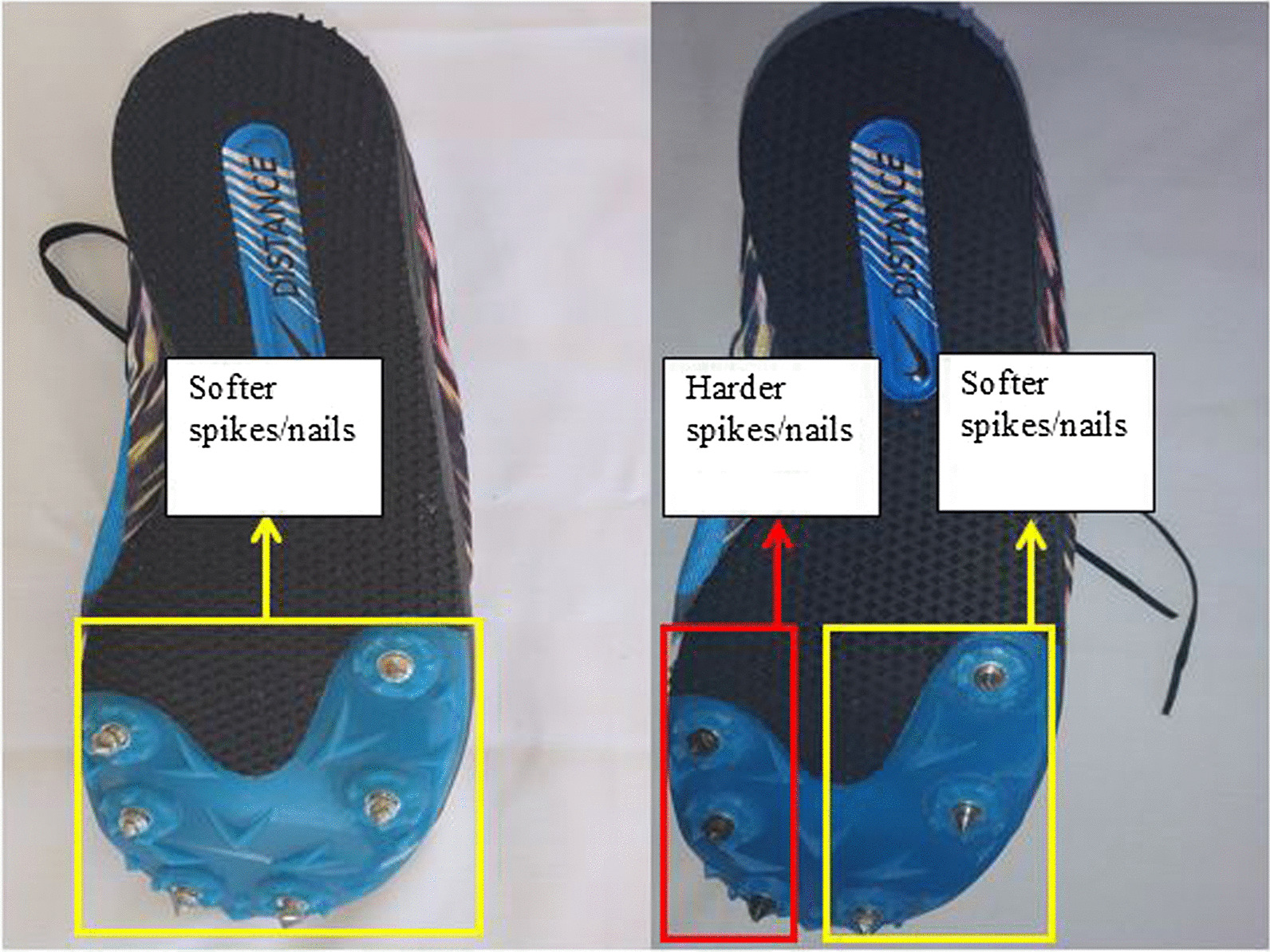
Single-stiffness (left side) and dual-stiffness (right side) spike distance running shoes used in this study

There is evidence that greater loading rates are related to a shorter time to peak impact vertical GRFs, which could increase the risk of sustaining running-related injuries [12]. For runners, it has previously been demonstrated that high lateral GRF result in pronation during running [13, 14]. Recent studies reported that free moments of the foot can be used as an index of torsional stress of the lower limbs [15, 16]. Free moments describe the vertical moment applied in the center of pressure, and have been associated with tibial stress fractures in distance runners [15]. Moreover, impact forces during running cause changes in the kinematic chain of the lower limbs [13, 14, 17, 18] such as altered peak rearfoot eversion angles which may result in increased stress of more proximal structures [19–21]. These biomechanical variables are important to define the etiology of running-related injuries and should be explored to describe potential instruments and/ or devices to reduce running-related injuries. Accordingly, dual-stiffness spike distance running shoe have been developed which are equipped with softer spikes/nails integrated in the lateral part of the sole and stiffer spikes/nails included in the medial part of the shoe sole (Fig. 1). There is preliminary evidence that this type of configuration reduces rearfoot pronation [22, 23]. However, it is unresolved whether dual- compared with single-stiffness spike distance running shoes has different effects on peak GRFs and their time to peak, average and instantaneous loading rates, free moments, and peak ankle eversion angles in runners with pronated feet.

Here, we aimed to examine the effects of running in dual- versus single-stiffness spike distance running shoes on GRFs, time to peak of impact vertical GRFs, average and instantaneous loading rates, peak free moment amplitudes, and peak ankle eversion angles in elite adult runners with pronated feet. With reference to the relevant literature [8, 22, 23], we hypothesized that running in dual- versus single-stiffness spike distance running shoes produces lower peak vertical GRFs, average and instantaneous loading rates, peak free moment amplitudes, and peak ankle eversion angles during running in elite adult runners with pronated feet.

## Methods

### Participants

Sixteen elite male long-distance runners who competed on a national level in either 5000 or 10,000 m race events volunteered to participate in this study. Elite male long-distance runners were recruited through online advertisements including a description of the inclusion/exclusion criteria and by directly approaching running clubs located around the university campus. Descriptive characteristics of the participants are shown in Table 1.

**Table 1:** Descriptive characteristics of the participants

Characteristics	Overall	5000 m runners	10,000 m runners
Sample size	16	8	8
Age (years)	28.2 ± 2.5	28.1 ± 2.6	28.3 ± 2.5
Body mass (kg)	72.3 ± 2.8	73.6 ± 2.7	71.0 ± 2.8
Body height (cm)	180.5 ± 8.9	180.2 ± 8.8	180.8 ± 8.9
Running experience (years)	11.2 ± 4.2	11.0 ± 4.2	11.4 ± 4.2
Personal best record (minute)	–	16.4 ± 0.4	33.5 ± 0.4

To be included in this study, participants had to have achieved personal best race times over 5- and/or 10-km races under 17 or 34 min during official running competitions. All participants were right-footed as determined by a kicking ball test. The health status (e.g., vascular complication, previous lower-limb injuries) of all included runners was assessed by an orthopedic surgeon prior to the start of the study. The inclusion criteria were: (i) free of lower limbs musculoskeletal injuries six months prior to the start of the study; (ii) training volume of at least 60 km per week; (iii) body mass index between 18 and 23 kg/m^2^; (iv) rearfoot eversion angle greater than 4° [24]; (v) navicular drop above 10 mm; (vi) a foot posture index above 10 [24]; and (vii) heel strike running pattern. The strike pattern was established through observation and using kinetic data [25]. In accordance with Bok et al. [26], the rearfoot eversion angle was determined as follows. First, subjects lay prone on a therapy table. Regardless of the calcaneal fat pad, the upper, middle, and lower bisection points of the calcaneus were marked and three points were connected to create a centerline. Thereafter, the subjects stood in a relaxed bipedal stance position with their feet apart as wide as an adult’s fist. The angle was measured between the centerline of the calcaneus and the vertical line to the ground [26]. The navicular drop was tested using a modification of the Brody method [27], with the subject in a weight-bearing position. Participants were asked to stand barefooted on a 4-in (10.16-cm) box, placing the entire body mass on the tested foot, while the other foot rested lightly on the box. The clinician palpated the medial and lateral aspects of the talar dome with the thumb and index finger placed just in front of the anterior aspect of the fibula and just anterior and inferior to the medial malleolus. The participant slowly inverted and everted the hindfoot and ankle until the depressions felt by the thumb and index finger of the clinician were equal. With the foot in this subtalar neutral position, the clinician measured the distance between the navicular tubercle and the floor in millimeters with a ruler. Thereafter, the participant was asked to completely relax the foot into full weight bearing, and the resulting position of the navicular was measured with the ruler. The clinician recorded the distance between the original height of the navicular and its final weight bearing position as the individual’s navicular-drop score. The Foot Posture Index consists of six items to quantify and classify foot posture [28]. These are (i) palpation of the head of the talus; (ii) curvatures above and below the lateral malleolus; (iii) position of the calcaneus in the frontal plane; (iv) prominence in the talonavicular joint; (v) the medial longitudinal arch’s congruence; and (vi) abduction/adduction of the forefoot. Each item was rated on a visual analogue scale ranging from − 2 to 2, resulting in a total score from − 12 to 12. Negative values indicate supinated foot posture and positive values indicate pronated foot posture. Foot Posture Index values ranged 10–12 classified as pronated feet [28]. The detailed description of the Foot Posture Index can be found elsewhere [28]. The exclusion criteria were: (i) a history of musculoskeletal surgery at the trunk and/or lower limbs; (ii) acute neuromuscular or orthopedic disorders (except foot pronation); and (iii) lower limbs length asymmetry above 5 mm [29]. Eligible participants provided written informed consent. The study conformed to the ethical guidelines of the latest version of Declaration of Helsinki and the procedures were approved by the Ethics Committee of the University of Mohaghegh Ardabili Iran (IR.ARUMS.REC.1398.408).

### Running shoes

Based on the availability at the local market, the following shoe model (Nike, Nike Zoom Rival, USA) was selected and adapted according to spike softness/stiffness (single versus dual-stiffness spike running shoes). In single-stiffness spike distance running shoes, we used regular (softer) spikes/nails in the medial and lateral part of the shoes (Fig. 1-left side). In dual-stiffness spike distance running shoes, we used regular or softer spikes/nails in the lateral part of the shoe and stiffer or harder spikes/nails in the medial compartment of the shoe (Fig. 1-right side). The mass of the shoes was similar for single and dual-stiffness spike running shoes and amounted to 310 ± 10 g. In other words, besides the reported differences in spike softness/stiffness, the running shoes were similar. The spike configuration in terms of soft and stiff nails was adapted by an expert sport shoe manufacturer in Ardabil city. The Vickers hardness test was used to assess spike/nail hardness value. Regular spike/nail and stiffer or harder spike/nail hardness were 190 ± 8 HV30/20 and 478 ± 9 HV30/20, respectively. Regular spikes/nails consisted of Iron [Fe] (98.41%), Manganese [Mn] (1.11%), Sulfur [S] (0.34%), and Silicon [Si] (0.14%) elements. Harder spikes/nails were made up out of Fe (98.60%), Mn (0.93%), and Si (0.47%) elements.

Eight days prior to the start of the study, each participant received a pair of single-stiffness (control) and dual-stiffness spike distance running shoes according to the foot size. The participating runners were kindly asked to get familiarized with the two running shoes by wearing the shoes during training on consecutive days (i.e., the single-stiffness spike distance running shoe on one day and the dual-stiffness spike distance running shoe on the next day) to allow familiarization with both shoe types.

### Overground running

Testing was always scheduled between 10:00 and 12:00 AM. Before testing, participants performed a standardized 10 min warm-up protocol consisting of jogging at low-to-moderate intensities for 7 min, followed by dynamic stretching for 3 min.

For the running trials, a 60 m walkway with a Bertec force plate (Bertec Corporation, Columbus, OH, USA) embedded in the middle of the walkway was used to collect GRFs data at 1000 Hz. The force plate was 60 cm long and 40 cm wide, and was oriented lengthwise in the running direction along the track. The plate was covered with a Mondo SuperX track surface. Accordingly, the surface was similar on top of the force plate and the surrounding running track. All participants were familiar with the laboratory situation and ran at a constant speed of 4.0 m/s [30] across the walkway. Six test trials were conducted per shoe condition. The shoe conditions were randomized and a 2 min rest between trials and a 5 min rest between shoe conditions was granted. Each subject received three familiarization trials to make sure that they ran at constant speed and actually hit the force plate with their dominant foot. A test trial was considered successful if running speed was 4.0 m/s ± 5%. Running time was monitored using a chronometer.

Objective criteria to discard a trial were: (i) the dominant foot did not land on the force plate; (ii) the participant lost balance during the trial; (iii) participants ran with a midfoot or forefoot strike pattern.

### Running kinetics

Kinetic data were processed according to a previous study [22]. Briefly, GRFs were low-pass filtered at 20 Hz (4th order Butterworth filter, zero lag). The heel strike and toe-off were identified using the force plate and a 10 N threshold (onset of force). GRFs during running, their time to peak, average vertical loading rates, and free moments have been reported to be among the most clinically relevant kinetic variables related to pathological gait/running patterns [22]. We extracted the first vertical peak force (Fz_HC_) from vertical GRFs data [22]. We calculated the positive (lateral) peak (Fx_HC_) from the medial–lateral curve, which occurs right after heel strike. These variables were chosen as the most relevant components based on previous research on GRF during running [25, 31–33]. GRF amplitudes were normalized to body weight (BW) and reported in %BW. A time to peak was defined as the time between the initial heel contact and the corresponding peak of the impact vertical component. Average vertical loading rates were computed as the average slope from 20 to 80% of the vertical GRF at the point of interest [22]. Instantaneous vertical loading rates were calculated as follows [34]:

$${{Instantaneous}}\;{{vertical}}\;{{ loading}} \;{{rate}} = \frac{{\Delta {{F}}_{{{{max}}}} }}{{\Delta {{t}}}} \quad {{where}} \;({{t}}_{20\% } < {{t}} < {{t}}_{80\% } )$$

here ΔF_max_ is the maximum change in vertical GRF, Δt is the time period between adjacent data points, t_20%_ corresponds to 20% of the time to peak impact, t_80%_ corresponds to 80% of the time to peak impact.

The free moment of the foot was computed as follows:

$${\text{FM}} = {\text{Mz}} + \left( {{\text{Fx}} \times {\text{COPy}}} \right) - \left( {{\text{Fy}} \times {\text{COPx}}} \right)$$

where: Mz is the moment around the vertical axis; x and y are the horizontal components of the center of pressure (COP), and Fx and Fy are the horizontal GRF components. Moreover, FM amplitudes were normalized with regards to BW × height. All running variables were averaged across six trials.

### Running kinematics

A three-dimensional motion analysis system (Vicon Nexus, Oxford Metrics, UK) was used to record the spatial position of markers on relevant body segments at a sampling frequency of 200 samples/s. Six complete force plate strikes of the dominant foot were registered. Since footwear can affect the distribution of loads on the joints in the lower quadrant [35], all reflective markers were directly placed on the skin of the relevant anatomical landmarks. The CAST marker set technique [36] was used whereby rigid clusters of four non orthogonal markers were attached over the lateral shank, the lateral thigh and the sacrum to track the segmental kinematics in six degrees of freedom. Four retroreflective markers (positioned over the 1st and 5th metatarsal heads, the most posterior aspect of the calcaneus and the most anterior tip of the toe) were attached to the control shoes with the foot being modelled as a rigid, single segment [37]. In visual 3D (C-Motion, Rockville, Maryland), joint kinematics were calculated using an X–Y–Z Euler rotation sequence equivalent to the joint coordinate system [38]. A trial was discarded if the dominant foot did not land on the force plate, if the participant targeted the platform, lost balance during the trial, ran with a mid or forefoot strike pattern, or even fell during running. Kinematic data were filtered using a 4th order low-pass Butterworth filter with a cutoff frequency of 10 Hz. The peak ankle eversion angle was calculated and used for further statistical analyses.

### Statistical analyses

Normality of data was examined and confirmed using the Shapiro–Wilk-Test. Accordingly, data were presented as means and standard deviations. Due to the within subject study design and normality of data, a paired sample t-test was computed. Effects sizes in the form of Cohen’s d [39] were computed using the following equation:

$$d = \left( {M_{1} - M_{2} } \right)/\left( {{\text{pooled }}\;{\text{standared}}\;{\text{deviation}}} \right)$$

In this equation, M_1_ and M_2_ stand for mean values for each shoe condition. Pooled standard deviation was computed using the following equation:

$${{Pooled}}\;{{ standard}}\;{{ deviation:}}\; \left({{{SD}}_{1} + {{SD}}_{2} } \right)/2$$

In this equation, SD_1_ and SD_2_ stand for standard deviation values for each shoe condition.

According to Cohen [39], d < 0.50 indicate small effects, 0.50 < d < 0.80 indicate medium effects, and d ≥ 0.80 indicate large effects. The significance level was set at *p* < 0.05. All analyses were performed using the Statistical Package for Social Sciences (SPSS) version 24.0.

## Results

No test-related injuries occurred during the study. Accordingly, data sets from all participants were included in the final analysis.

### Ground reaction forces

Paired t-tests revealed significantly lower Fx_HC_ (*p* = 0.021, d = 0.95) and Fz_HC_ (*p* = 0.010, d = 1.40) during running with dual- compared with single-stiffness spike running shoes (Table 2). Running with dual-stiffness spike running shoes resulted in significantly longer times to peak of Fz_HC_ (*p* = 0.004, d = 1.40) during running.

**Table 2:** Ground reaction forces and their time to peak at heel contact during running in single-stiffness and dual-stiffness spike running shoes

Variables	Components	Means ± SDs Single-stiffness spike shoes (Control)	Means ± SDs Dual-stiffness spike shoes	t	Sig	Effect size (d)
GRF (% BW)	Fx_HC_	13.76 ± 8.57	7.57 ± 4.42	2.578	0.021	0.95
	Fz_HC_	159.92 ± 19.83	129.48 ± 24.49	−2.874	0.010	1.40
TTP (% Stance time)	Fz_HC_	18.06 ± 6.36	23.75 ± 1.73	−3.405	0.004	1.40

*Fx*_*HC*_ peak lateral ground reaction force during heel contact, *Fz*_*HC*_ peak vertical ground reaction force during heel contact, *SD* standard deviation, *TTP* time to peak

### Loading rates and free moment amplitudes

**Fig. 2:**
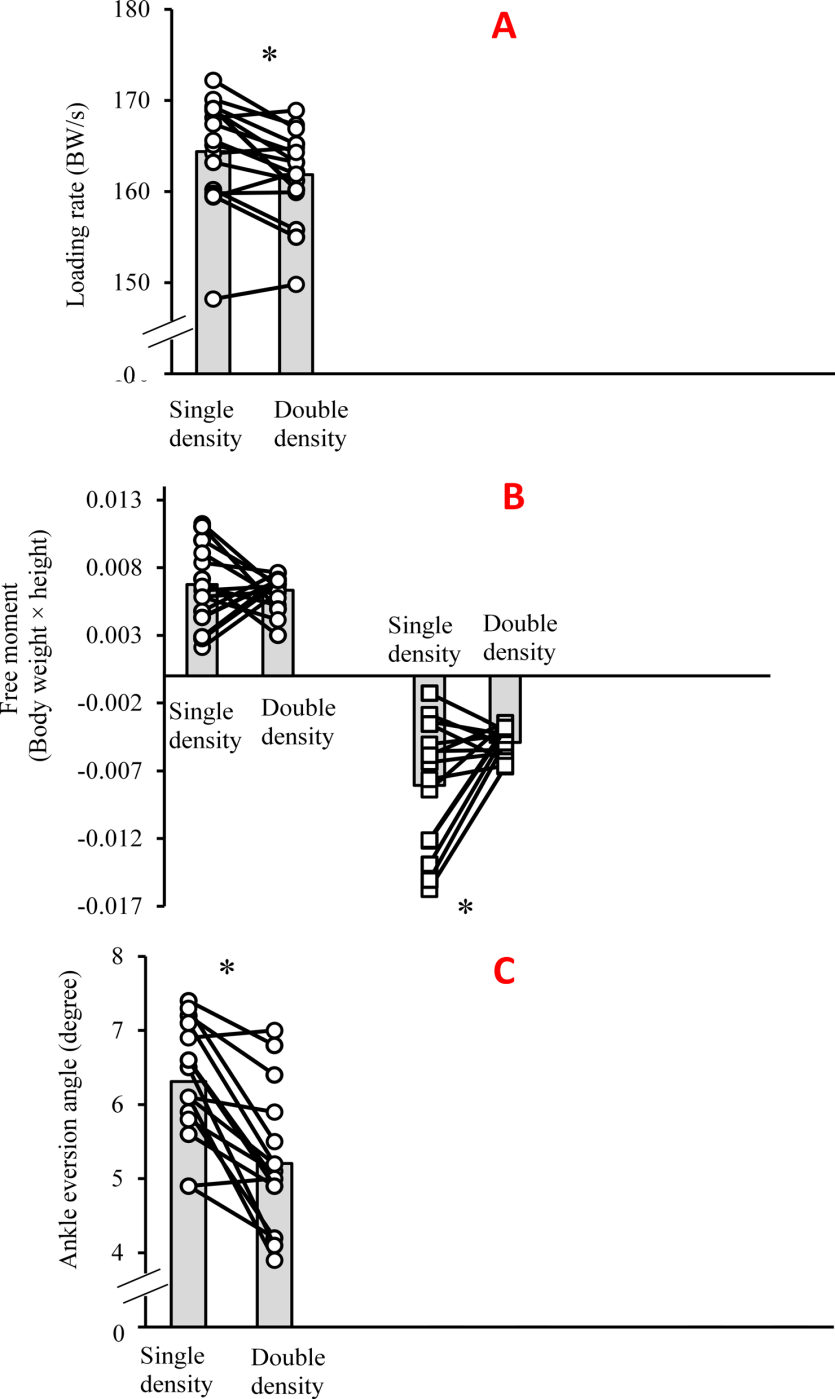
Individual and mean data of average loading rates (**a**), peak positive and negative free moments (**b**), and ankle eversion angles (**c**) when running in single-stiffness (control condition) versus dual-stiffness spike running shoes

The analysis revealed significantly lower average loading rates (*p* = 0.005, d = 0.46) and peak negative free moment amplitudes (*p* = 0.016, d = 0.81) when running with dual- compared with single-stiffness spike shoes (Fig. 2). Also, the analysis revealed significantly lower instantaneous loading rates when running with dual-stiffness (211.09 ± 6.31 BW/s) compared with single-stiffness (214.15 ± 6.07 BW/s) spike shoes (*p* = 0.021, d = 0.49).

### Peak ankle eversion angle

Finally, significantly lower peak ankle eversion angles were observed when running with dual- compared with single-stiffness spike distance running shoes (*p* < 0.001, d = 1.29) (Fig. 2).

## Discussion

This study examined the effects of running in dual- versus single-stiffness spike distance running shoes on ground reaction forces and their time to peak, loading rates, peak free moment amplitudes, and peak ankle eversion angles in elite male runners with pronated feet. The main findings of this study were that running in dual- versus single-stiffness spike distance running shoes resulted in lower peak lateral and vertical forces, lower average and instantaneous loading rates, and lower peak negative free moments and peak ankle eversion angles. Therefore, our hypothesis was confirmed.

### Ground reaction forces

This study showed that running in dual- versus single-stiffness spike distance running shoes resulted in lower peak lateral and vertical forces. For runners, it has previously been demonstrated that high lateral GRF result in over-pronation during running [13, 14]. Our results demonstrated that running in dual-stiffness spike distance running shoes significantly reduced peak lateral GRF. The large effect size indicates that this outcome is practically relevant*.* Notably, increased impact vertical GRF may constitute a mechanical risk factor for orthopedic injuries [40]. In this study, running in dual-stiffness spike shoes resulted in a significantly lower vertical impact peak force. With reference to the large effect size, this finding is practically relevant. This is the first study that provides preliminary evidence for the use of dual-stiffness spike distance running shoes in male runners with pronated feet. The mechanisms by which the change in density might have specifically reduced the magnitude of the vertical GRF peak may be due to changes in lower limbs muscle activities (e.g., tibialis posterior activity). However, we did not measure muscle activity in this study which is why this issue remains to be elucidated in future research. Besides the reported differences in nail softness/stiffness, the running shoes were similar. While single-stiffness spike shoes are characterized by regular (softer) spikes/nails in both, the medial and lateral sides of the shoe, dual-stiffness spike shoes have regular or softer spikes/nails in the lateral part of the shoe and stiffer or harder spikes/nails in the medial compartment of the shoe. Our findings together with the dual- / single spike shoe configuration suggest that the observed effects are due to differences in nail softness/stiffness. Findings from this study demonstrate that dual- compared with single-stiffness spike distance running shoes are effective for runners to maintain early vertical (Fz_HC_) and lateral (Fx_HC_) forces.

### Loading rates, free moment amplitudes and peak ankle eversion angles

This study showed that running in dual- versus single-stiffness spike distance running shoes resulted in significantly lower average and instantaneous loading rates. However, the small effect size indicates that this outcome may not be practically relevant which is why it should be verified in future studies. It has previously been demonstrated that repetitive loading during early stance phase results in subchondral bone microdamage associated with cartilage thinning [41]. The lower loading rate during running with dual-stiffness spike distance running shoes compared with single-stiffness spike distance running shoes is related to a longer period to reach peak vertical GRF at heel contact. Such timing adaptation may be associated with reduced peak ankle eversion angle as was described in our results.

The dual- versus single-stiffness spike distance running shoes resulted in significantly lower peak negative free moments for runners. It has previously been reported that negative free moments may indicate the torsional stress exerted on the lower extremities [42]. The leg’s excessive internal rotation is related to increased foot pronation [43, 44], and the foot muscles that control excessive pronation cannot be strong enough to counteract these forces from the hip and lower leg [45]. Further, it has been demonstrated that runners with a history of injuries (e.g., tibial stress fracture and pronation) showed greater free moment amplitudes than healthy (uninjured) runners [15, 46, 47]. This highlights the importance of assessing free moments and thus biomechanical loading of the lower extremities while running. Our study revealed that running in dual-stiffness spike distance shoes resulted in significantly lower peak negative free moments in runners with pronated feet. With reference to the large effect size, this finding is practically relevant.

This study has a few limitations that should be discussed. First, we included male elite runners only, which is why the outcomes of this study are specific to the population under investigation. Accordingly, they cannot be transferred to female runners or runners of different expertise levels. More research is needed in this area. Second, we did not record electromyographic activity in this study. Accordingly, we do not know how the neuromuscular system responded to the different shoe conditions. This should be done in future research. Third, we examined the acute effects of running with single- versus dual-stiffness spike distance running shoes. Future studies are needed that investigate the long-term effects of running in dual-stiffness spike distance running shoes on running mechanics. Fourth, it has to be established whether dual- compared with single-stiffness running shoes have similar effects on propulsion during running and/or sprinting. Finally, our study has not prospectively recorded injury rates. This should be realized in future studies.

## Conclusions

Running in dual- compared with single-stiffness spike distance running shoes resulted in lower loading rates, free moment amplitudes, and peak ankle eversion angles in male elite long-distance runners with pronated feet. Dual-stiffness spike distance running shoes are characterized by softer spikes/nails in the lateral part of the shoe and harder spikes/nails in the medial part of the shoe. Therefore, running shoes using dual-stiffness spikes appear to change running mechanics in male elite runners with pronated feet.

## Data Availability

The dataset generated and analysed during the current study are not publicly available in order to protect the individual privacy but are available, once the whole dataset is collected, from the corresponding author on reasonable request for researchers who have institutional review board/ethics approval and an institutionally approved study plan.

## Abbreviations

GRFs: Ground reaction forces
Fe: Iron
Mn: Manganese
S: Sulfur
Si: Silicon
Fz_HC_: First peak during heel contact
Fz_PO_: Second peak during the push-off phase
Fy_HC_: Braking force
Fy_PO_: Propulsion forces
Fx_HC_: Peak lateral ground reaction force
Fx_MS_: Peak lateral ground reaction force during mid stance phase
Fx_PO_: Peak medial ground reaction force during push-off phase
BW: Body weight
Mz: Moment around the vertical axis
COP: Center of pressure
